# Injectable Gelatin Hydrogel Suppresses Inflammation and Enhances Functional Recovery in a Mouse Model of Intracerebral Hemorrhage

**DOI:** 10.3389/fbioe.2020.00785

**Published:** 2020-07-14

**Authors:** Jiake Xu, Zhongxin Duan, Xin Qi, Yi Ou, Xi Guo, Liu Zi, Yang Wei, Hao Liu, Lu Ma, Hao Li, Chao You, Meng Tian

**Affiliations:** ^1^Neurosurgery Research Laboratory, National Clinical Research Center for Geriatrics, West China Hospital, Sichuan University, Chengdu, China; ^2^Department of Neurosurgery, West China Hospital, Sichuan University, Chengdu, China; ^3^Department of Integrated Traditional and Western Medicine, West China Hospital, Sichuan University, Chengdu, China; ^4^West China Brain Research Centre, West China Hospital, Sichuan University, Chengdu, China

**Keywords:** gelatin, hydrogel, intracerebral hemorrhage, inflammation, functional recovery

## Abstract

Intracerebral hemorrhage (ICH) is a devastating subtype of stroke with high morbidity and mortality. However, there is no effective therapy method to improve its clinical outcomes to date. Here we report an injectable gelatin hydrogel that is capable of suppressing inflammation and enhancing functional recovery in a mouse model of ICH. Thiolated gelatin was synthesized by EDC chemistry and then the hydrogel was formed through Michael addition reaction between the thiolated gelatin and polyethylene glycol diacrylate. The hydrogel was characterized by scanning electron microscopy, porosity, rheology, and cytotoxicity before evaluating in a mouse model of ICH. The *in vivo* study showed that the hydrogel injection into the ICH lesion reduced the neuron loss, attenuated the neurological deficit post-operation, and decreased the activation of the microglia/macrophages and astrocytes. More importantly, the pro-inflammatory M1 microglia/macrophages polarization was suppressed while the anti-inflammatory M2 phenotype was promoted after the hydrogel injection. Besides, the hydrogel injection reduced the release of inflammatory cytokines (IL-1β and TNF-α). Moreover, integrin β1 was confirmed up-regulated around the lesion that is positively correlated with the M2 microglia/macrophages. The related mechanism was proposed and discussed. Taken together, the injectable gelatin hydrogel suppressed the inflammation which might contribute to enhance the functional recovery of the ICH mouse, making it a promising application in the clinic.

## Introduction

Intracerebral hemorrhage (ICH) is a devastating subtype of stroke with high morbidity and mortality, However, there is no effective therapy method to improve its clinical outcomes to date (Krishnamurthi et al., 2013; Poon et al., 2014). Although the injury mechanism is complex and not fully understood, increasing evidence has shown that the inflammatory cascades are closely related with the progression of the injury such as activation of the microglia, the release of inflammatory cytokines, and neuron loss, and therefore inflammation could be regarded as one of the most important targets for the overall prognosis of ICH (Duan et al., 2019; Guo et al., 2019; Tschoe et al., 2020).

Recently, injectable hydrogels have attracted more and more research interests due to their innate merits, e.g., capable of minimally invasive implantation (Dimatteo et al., 2018; Wang C. et al., 2019), especially for stroke, since they can be injected initially as a fluid through a needle into the brain with stereotactic procedures, and then formed the gel upon crosslinking to irregularly shaped cavities at the implant site (Nih et al., 2016). In this context, there are some reports that injectable hydrogel has been used to promote host cell infiltrate and endogenous brain tissue repair (Ghuman et al., 2016), encourage angiogenesis and recovery of nerve circuits through guided drug and growth factor delivery (Nih et al., 2018), and also, injectable hydrogel could be applied to transplant stem cells to restore lost neurons (Hu, 2016). For brain implantation, like other implants, the immune response which partly depending on the characteristics of the implants such as biocompatibility significantly influences the interaction between the hydrogel and the surrounding host tissues (Tsui et al., 2019; Wissing et al., 2019). As a key innate immune cell in the brain, microglia is the most important defense against exogenous threats, where activated microglia/macrophages develop into two subtypes: pro-inflammatory microglia (M1, classically activated) and anti-inflammatory microglia (M2, alternatively activated) (Xiong et al., 2016), the polarization of which plays a crucial role in promoting the recovery of brain injury and nerve (Bai et al., 2020). Nevertheless, how to modulate the immune response and neuroinflammation through the implanted biomaterials such as hydrogel remains a great challenge to date.

In the case of hydrogel itself, a variety of different repair and anti-inflammatory cell pathways were induced through the binding of the implanted hydrogel to specific cell surface receptors(integrin mostly, which mediates the cell-signaling, the mutual recognition, and adhesion between cells and cells as well as cells and extracellular matrix) of endogenous brain cells via cell-adhesion peptides (Zhang et al., 2016; Rajkovic et al., 2018; Rowley et al., 2019; Michael and Parsons, 2020). For example, the arginine-glycine-aspartic acid (RGD) that binds to integrin is one of the mostly reported peptides (Huettner et al., 2018). However, previous study reported that RGD-modified hydrogel might result in the pathological angiogenesis (Li et al., 2017). In this regard, hydrogels based on natural proteins, such as collagen and gelatin or decellularized membranes, have the advantage of generally possessing the ligands necessary for cell adhesion. Gelatin derived from denatured and partly degraded collagen, and was widely used in the tissue engineering for its good biodegradability and biocompatibility, as well as adhesion to cells and lack of antigenicity (Lin et al., 2017; Mobaraki et al., 2019; Shi et al., 2019a, b; Zhang et al., 2019), and often used for cell encapsulation (Barthes et al., 2018), More importantly, gelatin retains cell adhesive motifs of RGD (Echave et al., 2017), a key biological functional sequence that could be used as an active target (Ge et al., 2018), promote angiogenesis and nerve regeneration (Li et al., 2017; Dursun et al., 2019; Samadian et al., 2020; Wu et al., 2020), reduce gliosis and accelerate neural progenitor cell migration (Nih et al., 2017; Motamed et al., 2019), influent inflammation (Zaveri et al., 2014; Nguyen et al., 2016), and elicit M2 polarization from macrophages *in vitro* (Cha et al., 2017; Wang et al., 2018; Kang et al., 2019) when binds to integrin receptor through ligand-receptor specific interactions. However, *in vivo*, we know that the interaction between host immunity and the implant depends on the microenvironment of adjacent tissue, resulting in a tissue-specific response to biomaterials (Taraballi et al., 2018; Feng et al., 2019; Wang Y. et al., 2019). Besides, unlike microglial or macrophage lines, the gene expression signature of microglia *in vivo* was shown to be unique (Butovsky et al., 2014), therefore it is inappropriate to apply the conclusions derived from macrophages or cell lines to microglia *in vivo*.

**FIGURE 1:**
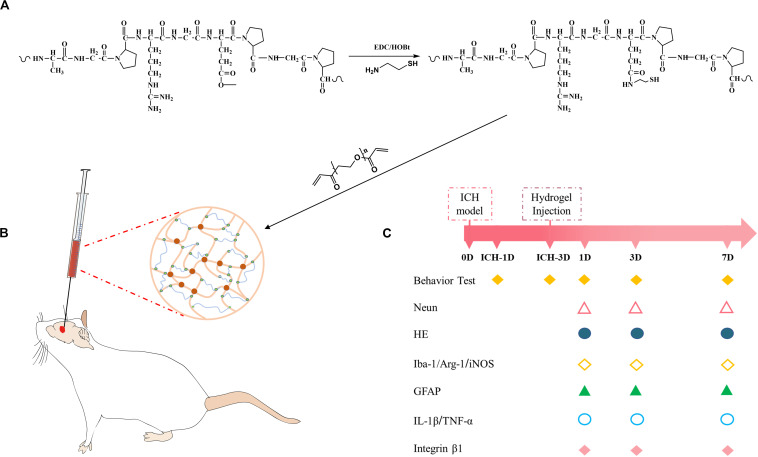
**(A)** Synthesis pathway of thiolated gelatin. **(B)** Schematic presentation of gelatin hydrogel injection into the lesion site in a mouse model of ICH. **(C)** Experimental protocol and timeline.

Herein, the possibility of modulating neuroinflammation using injectable gelatin hydrogel was explored. We hypothesized that gelatin hydrogel could specifically interact with brain immune cells through RGD-integrin and regulate the polarization of the immune cells, and thus suppressing the pro-inflammation and ameliorating the brain injury. To address this possibility, thiolated gelatin was first synthesized, and then the injectable gelatin hydrogel was prepared by Michael addition reaction (Figure 1A). The hydrogel was characterized by SEM, rheology, and cytotoxicity before an *in vivo* evaluation was performed in a mouse model of ICH (Figure 1B).

## Materials and Methods

### Materials

Gelatin (type B ∼ 225 bloom), *N*-(3-Dimethylaminopropyl)-*N’*-ethyl carbodiimide hydrochloride (EDAC), 5, 5′-dithiobis (2-nitrobenzoic acid) (DTNB), polyethylene glycol diacrylate (PEGDA, MW 6000 Da), cysteamine, and dithiothreitol (DTT) were purchased from Sigma (St. Louis, MO, United States). *N*-hydroxysuccinimide (NHS) was obtained from Pierce. Viability/Cytotoxicity Assay Kit was purchased from Invitrogen. Primary antibodies consisted of mouse anti-GFAP, rabbit anti-Iba-1, rabbit anti-iNOS, mouse anti-Arginase 1, and rabbit anti-neun were purchased from Proteintech (United States). Rabbit anti-integrin β1 was purchased from Huaan (China). Rabbit anti-IL-1β and TNF-α were obtained from Santa Cruz Biotechnology (United States). Secondary antibodies consisted of fluorescent Alexa 488 and 555 antibodies were purchased from Invitrogen (United Kingdom). Horse radish peroxidase (HRP-conjugated AffiniPure goat anti-rabbit) was obtained from Jackson (United States).

### Synthesis and Characterization of Thiolated Gelatin

The synthesis of thiolated gelatin is illustrated in Figure 1. The thiolated gelatin was synthesized according to a previous protocol with slight modification (Shu et al., 2003). Briefly, 1 g of gelatin was dissolved in 100 ml distilled water, and then cysteamine and EDAC were added as solids to the reaction with a molar ratio of -COOH/cysteamine/EDAC 1:2:2. The pH of the reaction solution was maintained at 4.75 by the addition of 1 M HCl. After 4 h the reaction was stopped by neutralizing the solution with 4 M NaOH. Five gram DTT was then added to the resulting solution and the pH of the solution adjusted to 8.5. After stirring for 8 h under N_2_, the pH of the solution was adjusted to 4.0 by the addition of 1 M HCl. The resulting solution was first dialyzed (3500 Daltons molecule weight cut-off) against HCl solution (pH 4.0) containing 100 mM NaCl under N_2_, followed by dialysis against HCl solution (pH 4.0) under N_2_. The solution was clarified by centrifugation, and the supernatant was sterilized with a 0.2 μm Millipore filter and then lyophilized. The degree of substitution (DS) of free thiols was determined using the Ellman method. The structure of thiolated gelatin was characterized by ^1^H NMR spectrum in D_2_O.

### Preparation of Injectable Gelatin Hydrogel

PEGDA was dissolved in PBS to obtain a 10% (w/v) solution. Thiolated gelatin was dissolved in PBS. For the preparation of the hydrogel, the PEGDA solution was added to the thiolated gelatin solution with a molar ratio of acrylate/thiol 1:2 to prepare hydrogel precursor solution and initiate crosslinking, in which the final concentration of thiolated gelatin was 3% (w/v). Gelation time was determined by a test tube inverting method.

### Scanning Electron Microscopy (SEM) and Porosity Measurement

SEM was used to observe the morphology of the freeze-dried hydrogel. After coating with gold, the cross-sectional morphologies were viewed with a JSM-6390LV SEM. The porosity of the hydrogels was measured according to the literature (Tian et al., 2012). Briefly, the freeze-dried hydrogel was immersed in ethanol under vacuum for 20 min and then taken out to be weighed after absorbing the excess of ethanol with filter paper. The porosity was calculated according to the following equation:

(1) Porosity = [(W1−W2/ρV] ×100%

where W1 and W2 are the weights of the hydrogel before and after immersion in ethanol, respectively, ρ is the density of ethanol and V is the volume of the hydrogel.

### Rheological Test

The storage and loss modulus were measured with a plate-to-plate rheometer (MCR 302, Anton Paar, Ashland, VA, United States) using a 25 mm plate under a constant strain of 1% and frequency of 10 rad/s at 37°C.

### Cytotoxicity of the Hydrogel

For assay the cytotoxicity of the hydrogel, the primary rat MSC cells were encapsulated in the hydrogel with a cell density of 1 × 10^5^ cells per 100 μl of the hydrogel precursor solution. After 1 and 5 days of culture, the viability of the encapsulated cells was determined using the live/dead viability/cytotoxicity assay kit from Invitrogen. Briefly, 1 μM of ethidium homodimer-1 and 0.25 μM of calcein acetoxymethyl ester from the kit were diluted with 500 μl DMEM without phenol red. The hydrogels were stained for 30 min at room temperature in the dark and imaged with confocal microscopy (Nikon A1R + MP, Japan). Green (live cells) and red (dead cells) fluorescence images were collected separately and merged to determine cell viability as the ratio of viable cells to total cells counted.

### Animals and ICH Model

The animal experimental protocols were approved by the Animal Ethical Committee of Sichuan University. Twenty four adult Kunming (KM) mice (2–3 months old and weighing 22–28 g, Dashuo Laboratory Animal Co. Ltd, China) were housed under a 12/12 light/dark cycle conditions with free access to food and water for this study.

The ICH modeling in mice was induced by intracranial injection of type VII collagenase. Baseline weight was recorded for each mouse before the experiment. The mice were anesthetized intraperitoneally by the injection of 10% chloral hydrate (30 μl/10 g) and placed in a stereotactic frame (RWD life science, China). A burr hole of 1mm diameter was drilled in 0.8 mm anterior and 2.0 mm lateral (right) of bregma, and then injected with collagenase type VII (0.075 Units in 0.5 μl saline; Sigma, United States) into the right basal ganglia region from the hole (2.9 mm depth below the surface of the skull) at a rate of 0.1 μL/min. After injection, the needle was left in the place for 10 min before withdrawal, and the skull hole was closed with bone wax. Finally, the wound was sutured, and the animal was placed in a warm box with free access to food and water.

### Gelatin Hydrogel Injection

Three days post-ICH with the hematoma size became stable (Yang et al., 2017), mice were subjected to the gelatin hydrogel injection procedure by placing in a stereotactic frame with 10% chloral hydrate (30 μl/10 g, i.p.) anesthetized, and the body temperature maintained at 37°C using the heating pad. After removing the bone wax covering the cranial window, the lesion site was exposed. The hydrogel precursor solution was loaded into a microsyringe. For the ICH + Hydrogel group (*n* = 12, 4 mice per time point), mice were injected 4 μl precursor solution at a rate of 1 μl/min to the lesion using the previous coordinates. The needle was left in the place for 10 min to allow the solution to gel before removing it from the brain slowly, and the skull hole was closed again with bone wax followed by suturing the wound. In the ICH + Vehicle group (*n* = 12, 4 mice per time point), mice were injected with the same volume of PBS as control.

### Body-Weight Change and Neurobehavioral Testing

To assess the body-weight change and neurobehavior of the mice, an investigator blinded to two groups evaluated the mice with corner turn and seven neurological deficits tests on day 1 and 3 after the ICH modeling and day 1, 3, and 7 after gelatin hydrogel and vehicle injection, and measuring the weight simultaneously. For the corner turn test, the mice were placed between the two boards facing a 30°corner. When mice entering deep into the corner, both sides of the vibrissae are stimulated together, healthy animals tend to turn left or right randomly, while animals with unilateral brain damage tend to turn to the ipsilateral side. Twenty tests were repeated in each testing day with at least 30 s interval time between two tests, and the right turn percentage was calculated as right turns/all turns (Zhang et al., 2002). Neurological deficits tests include body symmetry, gait, climbing, circling behavior, front limb symmetry, compulsory circling, and whisker. Each test was graded from 0 to 4, and the maximum deficit score of 28 (Hazel, 1998).

### Histology and Immunostaining

#### Histological Treatment

Mice were deeply anesthetized by an overdose of 10% chloral hydrate and sacrificed via transcardial perfused with 4% paraformaldehyde (PFA) in 0.1 M phosphate buffer saline (PBS, pH = 7.4). The brain tissues were collected and post-fixed in 4% paraformaldehyde for 24 h, and then the tissues were trimmed as appropriate size (3 mm anterior and posterior to the bregma) for paraffin embedding and cut into 3-μm-thick coronal sections with a microtome (Leica RM2235, Germany). The slides were dried on a warmer at 60°C for 12 h. Before the staining, each brain sections were deparaffinized with pure xylene(three times) for 15 min each, then rehydrated in alcohol gradient (100% to 70%, 10 min each) and washed with distilled water.

#### Hematoxylin and Eosin Staining

Routine hematoxylin and eosin (H&E) staining was conducted for the perihematomal morphological changes observing and showing the host tissue-gelatin hydrogel interface. After the deparaffinized, the paraffin sections stained with hematoxylin for 5 min, rinsed under the tap water for 30 s, then put sections into 1% hydrochloric acid ethanol differentiation liquid solution for 5 s, and rinsed water for 5 min. Next, the eosin dye was redyed for 30 s, washed with distilled water for 10 s. After dehydrated with graded ethanol, the sections were mounted with mounting medium. Histologic sections were observed with a light microscope (BX43; Olympus, Tokyo, Japan).

#### Immunostaining

After the deparaffinized, antigen retrieval was achieved by microwave in EDTA buffer (PH9.0) for 20 min, cooled at room temperature. For immunofluorescence staining, sections were incubated in 10% blocking sera for 40 min prior at 37°C (Endogenous peroxidase activity was blocked with 0.6% hydrogen peroxide for immunohistochemical), then washed in 0.1 M PBS again, and incubated with primary antibody overnight at 4°C. Primary antibodies consisted of mouse anti-GFAP (1:500 dilution) to visualize the glial scar, rabbit anti-Iba-1 (1:200 dilution) to detect microglia/macrophages, rabbit anti-iNOS (1:200 dilution) and mouse anti-Arginase-1 (1:200 dilution) to detect different subtypes of Microglia/macrophages, rabbit anti-Neun (1:200 dilution) to detect surviving neurons, rabbit anti-integrin β1 (1:100 dilution) to detect the expression of integrin β1, and rabbit anti-IL-1β and TNF-α (1:200 dilution) to quantify the level of inflammation with immunohistochemical. After rinsing and washing with 0.1 M PBS three times for 5 min, sections were incubated with a secondary antibody for 30 min at 37°C and washed in PBS. Secondary antibodies consisted of appropriate fluorescent Alexa 488 or Alexa 555 antibodies (1:500 dilution). The immunofluorescence staining sections were observed through a fluorescence microscope (AX10 imager A2/AX10 cam HRC; Carl Zeiss, Germany). For immunohistochemistry, the sections were incubated with secondary antibody HRP for 30 min at 37°C and followed by washing in PBS. The color was visualized using peroxidase reaction with 3′,3′-diaminobenzidine, and then observed under a light microscope (BX43; Olympus, Tokyo, Japan).

### Image Analysis

The results were measured and evaluated by a blinded observer with the open-source software ImageJ/Fiji (US National Institutes of Health^1^). Three sections per mouse and three randomly selected microscopic fields per section around the hematoma area were used for quantitative analysis. The number of M1 microglia (iNOS^+^/Iba1^+^ cell), and M2 microglia (Arginase-1^+^/Iba-1^+^ cells) were evaluated by cell counts, then the ratio to Iba-1^+^ cells represents the percentage of microglia of different subtypes (%). Neuron density is expressed as the ratio of the number of Neun^+^ cells in the microscopic field to the area of the microscopic field (/mm^2^). The IL-1β, TNF-α, Iba-1, and GFAP positive areas around hematoma were quantified in each field to assess the expression level of IL-1β and TNF-α, the activation levels of microglia and astrocytes, respectively. Fluorescence stained image of integrin β1 was constant-thresholded using ImageJ/Fiji program to subtract background staining, then the fluorescence intensity in each cell area was calculated.

### Statistical Analysis

All data were expressed with mean ± standard error of the mean. Comparison of means between two groups was analyzed by the Student’s *t*-test, and statistical evaluations were performed using the GraphPad Prism 6.0. *P*-value was set at 0.05 for statistical significance.

## Results and Discussion

### Synthesis of Thiolated Gelatin

Thiolated gelatin was synthesized through EDC chemistry as shown in Figure 1A, where cysteamine was coupled to the gelatin carboxylates at pH 4.75, at which carbodiimide nitrogens appear to be sufficiently protonated while gelatin mainly presents as the carboxylate. The structure of the final product was characterized by ^1^H NMR spectrum in D_2_O. As shown in Supplementary Figure S1, there are two new peaks that appeared, one is at 2.8 ppm corresponding to hydrogen of methylene close to thiol, and the other is at 2.6 ppm that assigned to hydrogen of methylene adjacent to amide. The content of thiol in thiolated gelatin was determined by Ellman method, and the results showed that the product has a thiol content of 0.48 mmol/g, which corresponds to 39.2% DS.

### Preparation and Characterization of Injectable Gelatin Hydrogel

**FIGURE 2:**
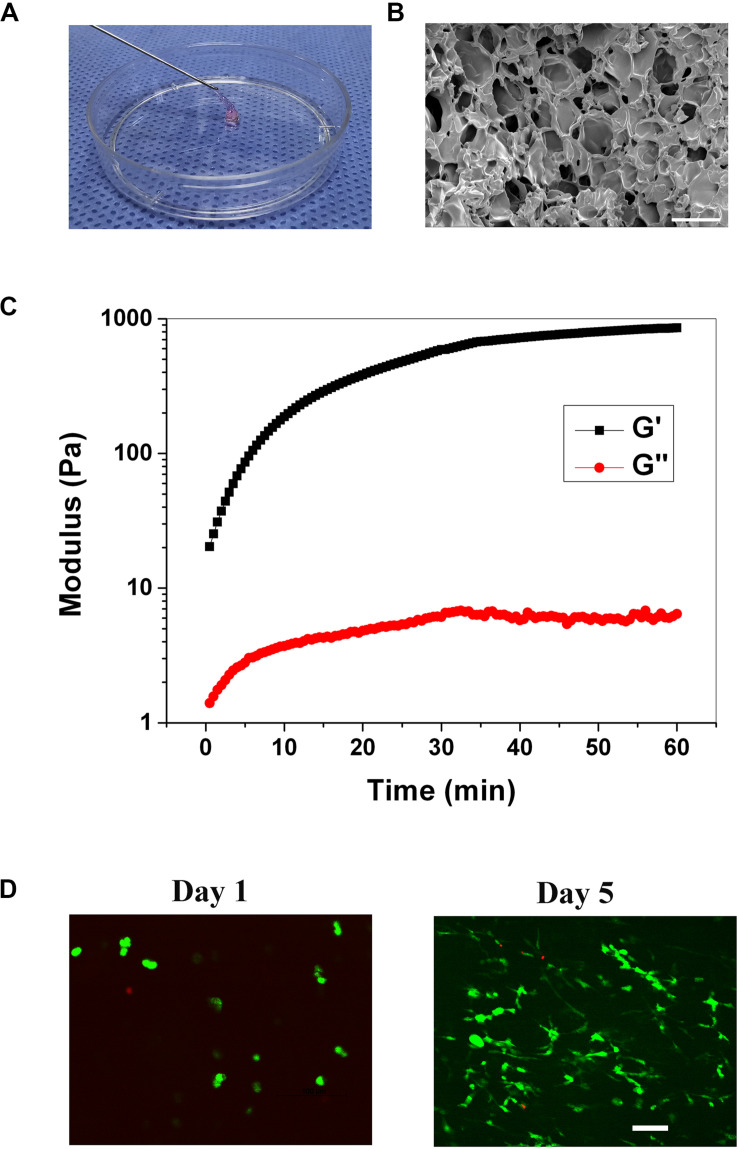
**(A)** Injection operation of the gelation hydrogel (staining with phenol red). **(B)** SEM image of the hydrogel, scale bar = 100 μm. **(C)** The rheological curve of the hydrogel. **(D)** Live/dead staining of the cells within the hydrogel on day 1, and 5, scale bar = 100 μm.

The injectable gelatin hydrogel was prepared by Michael addition reaction between the thiols in the thiolated gelatin and acrylates in the PEGDA to form a three-dimensional network under physiological conditions. The molar ratio of thiol relative to acrylate was set at 2/1 to ensure that no unreacted and potentially cytotoxic electrophiles remain in the hydrogel. The gelation time of the hydrogel was 6.5 ± 1.3 min, which was in the range of the clinical operation time (5–15 min). Within the gelation time, the injectable operation of the hydrogel was shown in Figure 2A. To study the pore structure of the hydrogel, SEM was carried out on the cross-sectional morphology of the freeze-dried hydrogel. As shown in Figure 2B, the cross-sectional of the hydrogel exhibited a three-dimensionally interconnected pore structure, with a pore size of 30–100 μm. The porosity of the hydrogel was 88.8 ± 2.5% when measured by the liquid displacement method. Thus, based on SEM and porosity measurements, it was reasonable to assume that the hydrogel would be beneficial for cell infiltration and the exchange of nutrients and metabolites. The storage (G’) and loss modulus(G”) were measured with a plate-to-plate rheometer. The maximum G’ for the hydrogel was 857 Pa (Figure 2C), which was in the range of the modulus of the brain tissue (100–1000 Pa), indicating that the hydrogel was sufficiently soft and might be compatible with brain tissue (Engler et al., 2006; Budday et al., 2017). Prior to the *in vivo* study, cytotoxicity of the hydrogel was evaluated using live/dead staining. As shown in Figure 2D, on day 1, the cell exhibited round morphology, whereas extensive spreading was observed on day 5, indicating that the RGD sequences within the hydrogel promoted the cell adhesion. The cell viability was 92.3 and 95.1% on day 1 and 5, respectively, both of which indicated that the hydrogel was compatible with the cells *in vitro*.

### Body-Weight Change and Functional Recovery After Gelatin Hydrogel Injection

**FIGURE 3:**
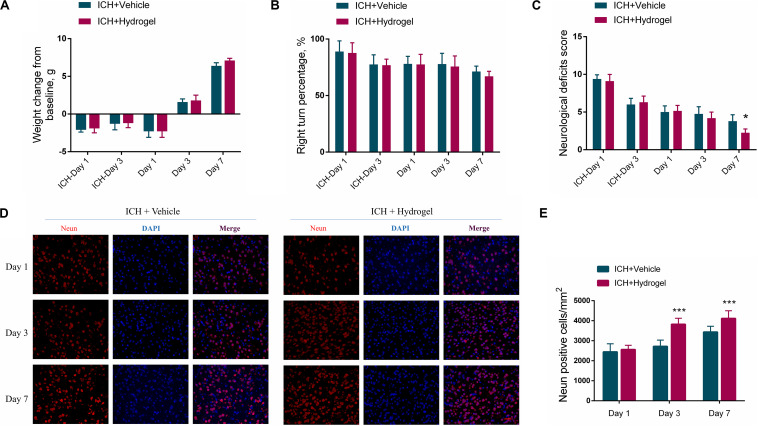
**(A)** Body-weight change. **(B)** The statistical graph of the corner turn test. **(C)** The statistical graph of the neurological deficit testing. **(D)** Representative images for Immunofluorescence staining of neuron marker Neun, scale bar = 20 μm. **(E)** The statistical graph of the Neun positive cells. Data are present as mean ± SD (*n* = 4), *P < 0.05, ***P < 0.001.

In consideration of the potential side effects after the hydrogel injection, the body-weight change and neurobehavior of the mice were monitored. As shown in Figure 3A, after ICH modeling (ICH-Day 1, and 3), the mice had a weight drop from the baseline. When the hydrogel or vehicle injection, the body-weight of the mice remained below the baseline on day 1, while they are recovering with time prolonged, with body-weight steadily increased on day 3 and 7. No significant variation had been detected between the two groups at each time point, implying no overt adverse effects of the hydrogel.

In the corner turn test, the percentage of right turn had a gradual decline in both two groups, and there was no statistical difference between them in each time point (Figure 3B). In the neurological deficit testing (Figure 3C), scores of both two groups were highest on the first day after ICH and descended with time. Recovery in the ICH + Hydrogel group was quicker than that in the ICH + Vehicle group and showed a significant difference on day 7 (4 ± 1 vs. 2 ± 1, *P* < 0.05). Just as tests differ in their selectivity and sensitivity for various deficits, neurological deficit scores mainly assess a variety of motor, sensory, reflex, and balance responses (Schaar et al., 2010), which is sensitive to nerve function deficit in the acute stage. In contrast, the corner turn test mainly focuses on the sensorimotor and postural asymmetry after ICH (Zhang et al., 2002). In our study, the assessment period was within 7 days and thus the neurological deficit test appears more sensitive to monitor the neurobehavior of the mice.

To explain why the ICH + Hydrogel group exhibited an enhanced functional recovery in comparison with the ICH + Vehicle group, the neuron density around the lesion was investigated since it plays an important role in motor function recovery after ICH (Miao et al., 2018; Chen et al., 2019). Figures 3D,E show the immunofluorescence staining and the statistical analysis of the neuron marker Neun, which, as expected, indicated that the hydrogel injection significantly rescued ICH-associated neuron loss, and had higher neuron density than that in ICH + Vehicle group on day 3 (2703 ± 311 cells/mm^2^ vs. 3844 ± 297 cells/mm^2^, *P* < 0.001) and 7 (3436 ± 290 cells/mm^2^ vs. 4117 ± 384 cells/mm^2^, *P* < 0.001).

### The Overall Change of the Gelatin Hydrogel After Injection

**FIGURE 4:**
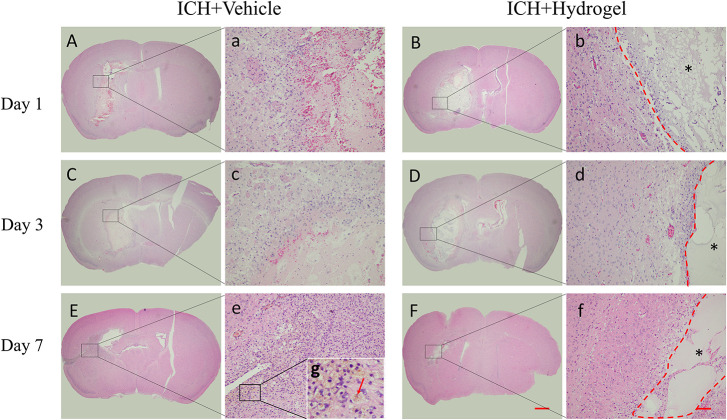
H&E staining. **(A–F)** The overall morphology of the injured brain in different time-point after gelatin hydrogel injection, scale bar = 500 μm. **(a–f)** Magnified images show the host tissue-lesion or gelatin hydrogel interface, scale bar = 50 μm. **(g)** Local magnification image of hemosiderin deposition. *Represents the hydrogel.

The H&E staining of the brain sections as shown in Figure 4. In both two groups, the lesion site consisted of a large amount of necrotic debris on the first day (Figures 4A,B). With the time prolonged, the lesion areas decreased on day 3 and 7. In the ICH + Vehicle group, there are many inflammatory cells surrounding and infiltration the lesion, and then a scar formed with hemosiderin deposition on day 7 (Figures 4E,e, red arrow). On the contrary, in the ICH + Hydrogel group, there is less inflammatory cell appearance and the hydrogel filled the lesion and was evenly spread throughout the entire stroke cavity over time, without dense glial scar surrounding (Figures 4B,D,F). Interestingly, some cells are infiltrating into the hydrogel on the first day. This phenomenon is similar to the Ghuman’s (Ghuman et al., 2018) study, who suggested that these cells are not a brain origin, but likely an immune origin (Modo, 2019), which was responding quickly to the damaged tissue.

### Gelatin Hydrogel Injection Inhibited the Activation of Microglia/Macrophages and Astrocytes

**FIGURE 5:**
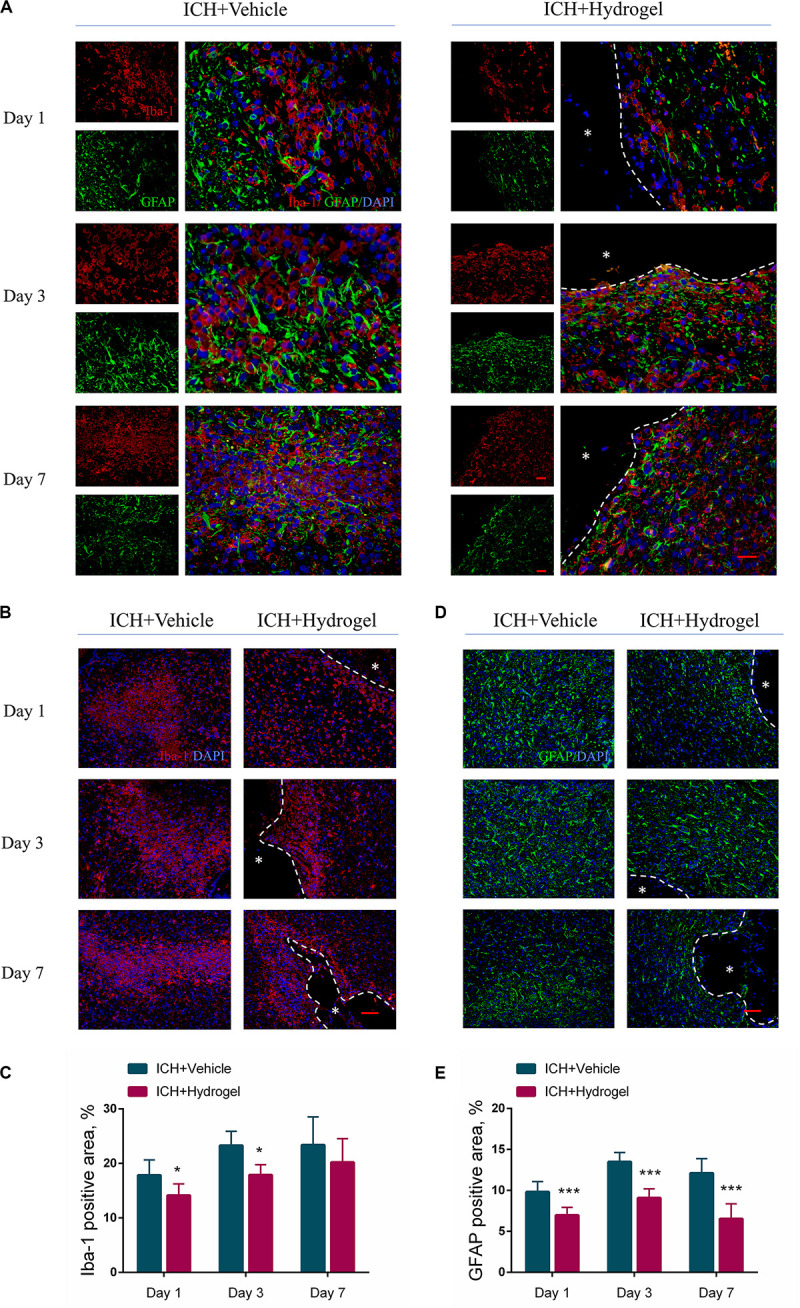
Gelatin hydrogel affects the activation of microglia/macrophages and astrocytes. **(A)** Double immunofluorescence staining was performed with astrocyte marker GFAP (green) and microglia/macrophages marker Iba-1 (red) in brain sections, scale bar = 20 μm. **(B)** Representative images for Immunofluorescence staining of Iba-1, scale bar = 50 μm. **(C)** The percentage of Iba-1 positive area analysis. **(D)** Representative images for Immunofluorescence staining of GFAP, scale bar = 50 μm. **(E)** The percentage of GFAP positive area analysis. *Represents the hydrogel. Data are mean ± SD, *n* = 4 mice per group, *P < 0.05, ***P < 0.001.

Microglia/macrophages and astrocytes are abundant around the lesion site, which defines the boundary between the normal tissue and the injured area, and thus Iba-1 and GFAP were stained to investigate the activated microglia/macrophages and astrocytes over time. As shown in Figure 5A, in ICH + Vehicle group, the activated microglia/macrophages and astrocytes gradually developed a structurally disordered scar structure around the lesion as time went on, while in the ICH + Hydrogel group, commonly the cells with the deepest infiltration distance on day 1 and 3 are activated microglia/macrophages and followed by astrocytes. This chain-like path formed might allow other cells to pursue their infiltration (Ghuman et al., 2016). On day 7, the invading microglia/macrophages and astrocytes were evenly distributed around the remaining hydrogels.

For the activated microglia/macrophages, the Iba-1 positive area in both two groups increased from day 1 to 7 (Figure 5B), and there were more activated microglia/macrophages in ICH + Vehicle group at each time point than that in the ICH + Hydrogel group with a significant difference on day 1 (17.84 ± 2.81% vs. 14.15 ± 2.09%, *P* < 0.05) and 3 (23.30 ± 2.58% vs. 17.90 ± 1.86%, *P* < 0.05) (Figure 5C), indicating that the activation of the microglia/macrophages was suppressed in the ICH + Hydrogel group. This phenomenon may be related to the presence of the RGD adhesion motif in the hydrogel as previously reported (Nih et al., 2017).

In the case of the activated astrocytes, as illustrated in Figure 5D, astrocyte activation increased rapidly in both two groups from the first day, and reached the peak on day 3, followed by a decline on day 7, and the activated astrocytes in the ICH + Hydrogel group at each time point were significantly less than that in the ICH + Vehicle group consistently (Figure 5E, *p* < 0.001), which means the hydrogel injection can inhibit astrocyte activation. In general, loss of neurons in the core of the lesion will be replaced by the glial scar eventually, while if it’s displaced by the hydrogel, the glial scar would be potentially decreased, which has been observed previously as attributing to the biomaterial-glial scar interaction (Ghuman et al., 2016, 2018; Gorenkova et al., 2019).

Glial scar, the final form of reactive astrogliosis after stroke, on the one hand, has been thought to be detrimental to neuronal growth, preventing axonal regrowth in chronic stages since the mid-20th century (Cregg et al., 2014), however, a recent study revealed that relieving glial scar was failed to result in axonal regeneration after spinal cord injury, which did not address the effect of scar ablation on other cells in the damaged CNS (Anderson et al., 2016; Adams and Gallo, 2018). So, it remains difficult to determine what the confounding factor is. On the other hand, activated astrocytes are neuroprotective by limiting the inflammatory response and restricting the immune cascade of the damaged tissues in the acute phase of stroke (Doyle et al., 2008). Thus in our conditions further studies are required to determine the effect of the interaction between the hydrogel and the glial scar on this process.

### Gelatin Hydrogel Injection Modulated Microglia/Macrophages Polarization

**FIGURE 6:**
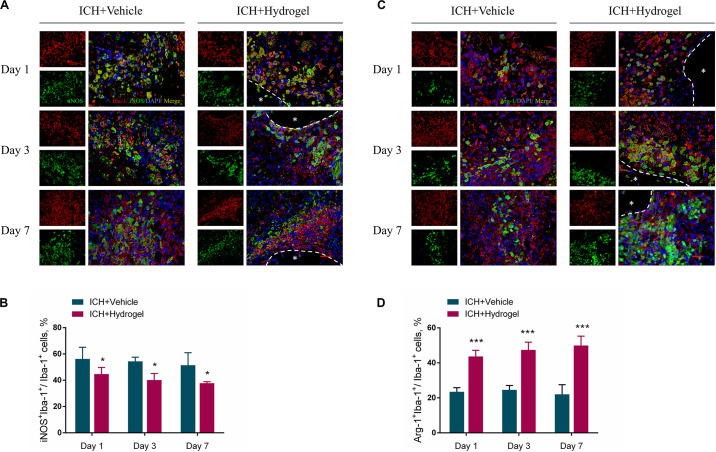
Effects of gelatin hydrogel on microglia/macrophages polarization. **(A)** M1 pro-inflammatory microglia/macrophages marker iNOS^+^Iba-1^+^. **(B)** The percentage of iNOS^+^Iba-1^+^cells/Iba-1^+^cells in two groups. **(C)** M2 anti-inflammatory microglia/macrophages marker Arg-1^+^Iba-1^+^. **(D)** The percentage of Arg-1^+^Iba-1^+^ cells/Iba-1^+^cells in two groups. Scale bar = 20 μm. Data are mean ± SD, *n* = 4 mice per group, *P < 0.05, ***P < 0.001.

To determine the effect of the hydrogel on the microglia/macrophages polarization, the expression of nitric oxide synthase 2 (iNOS) and Arginase1(Arg-1) was chosen to identify M1 and M2 phenotype, respectively (Lan et al., 2017). Figures 6A,C respectively show the double immunofluorescence staining of iNOS/Iba-1 and Arg-1/Iba-1 on day 1, 3, and 7 after hydrogel injection. Figure 6B revealed that the percentage of M1 cells peaked on day 1 in both two groups and then fell with time. Meanwhile, ICH + Hydrogel group showed a significantly lower percentage of M1 cells than that in the ICH + Vehicle group on day 1 (56.25 ± 8.84% vs. 44.68 ± 5.04%, *P* < 0.05), 3 (54.39 ± 3.16% vs. 40.14 ± 5.05%, *P* < 0.05), and 7 (51.41 ± 9.57% vs. 37.74 ± 1.18%, *P* < 0.05). On the contrary, the percentage of M2 cells in the ICH + Vehicle group maintained the similar level on day 1 and 3 and had a slight decline on day 7, while it was shown a steady increase in the ICH + Hydrogel group, and there were significant differences between the two groups at each time point (Figure 6D, day 1: 23.46 ± 2.38% vs. 43.65 ± 3.54%, *P* < 0.001; day 3: 24.59 ± 2.47% vs. 47.44 ± 4.41%, *P* < 0.001; day 7: 22.01 ± 5.49% vs. 49.92 ± 5.35%, *P* < 0.001).

The mechanism by which biomaterials enable microglia/macrophages polarization into the anti-inflammatory phenotype remains largely unknown. Gelatin retains the cell adhesive motifs-RGD, which has been reported to elicit anti-inflammatory effects from macrophages *in vitro* as well as increase cellular adhesion (Lynn et al., 2010; Zaveri et al., 2014; Cha et al., 2017; Wang et al., 2018; Kang et al., 2019). Besides, osteopontin containing RGD can significantly suppress the inductions of iNOS in postischemic brains, demonstrating anti-inflammatory effects (Jin et al., 2016). Consistently, our results demonstrate that the injected gelatin hydrogel in the ICH cavity can inhibit the M1 polarization while promoting anti-inflammatory M2 phenotype, which may have potential promising to modulate inflammatory response after ICH.

### Gelatin Hydrogel Injection Reduced the Release of IL-1β and TNF-α

**FIGURE 7:**
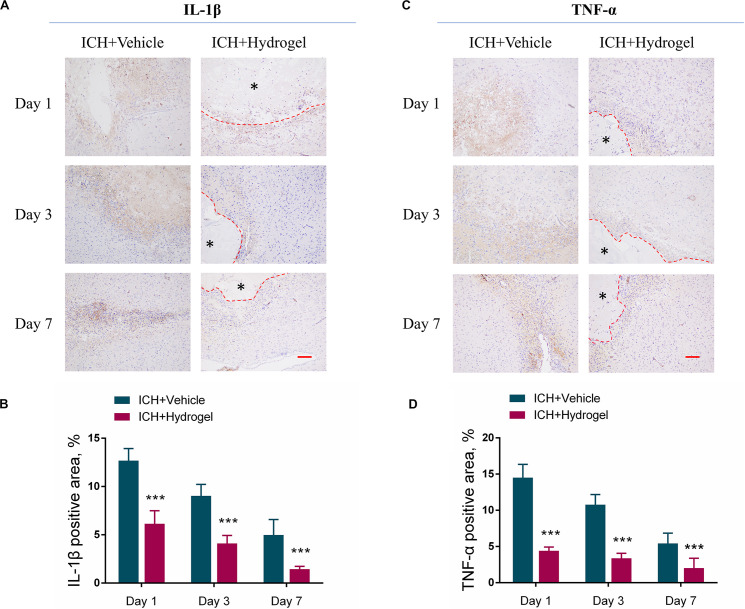
Effects of gelatin hydrogel on pro-inflammatory cytokine. **(A)** Immunohistochemical staining of IL-1β around the lesion. **(B)** The area percentage of IL-1β. **(C)** Immunohistochemical staining of TNF-α around the lesion. **(D)** The area percentage of TNF-α. Scale bar = 50 μm. *Represents the hydrogel. Data are mean ± SD, *n* = 4 mice per group, ***P < 0.001.

Pro-inflammatory cytokines, such as interleukin-1β (IL-1β), and tumor necrosis factor-α (TNF-α), play an important role in maintaining cellular function and inflammatory activation (Möller and Villiger, 2006; Zhou et al., 2019; Gao et al., 2020). Nevertheless, it is harmful if the cytokines keep working throughout inflammation. After ICH, microglia were activated and various circulating immune cells immediately entered the brain, then the chemokines, pro-inflammatory cytokines (IL-1β and TNF-α) and other immune molecules were released (Wang and Doré, 2007) which further activated resident and migrating immune cells, and caused a continued cycle of the inflammatory response, the process of which increased the brain cell death produced inflammatory injury to the surrounding brain tissue (Tschoe et al., 2020). To investigate whether the hydrogel injection has the anti-inflammatory effect, the expression of pro-inflammatory cytokines such as IL-1βand TNF-αwas determined by immunohistological staining. As shown in Figures 7A–D, the expression of both IL-1β and TNF-α was decreased over time in two groups, and the ICH + Hydrogel group showed significantly lower of both two pro-inflammatory cytokines than that in the ICH + Vehicle group (*P* < 0.001), indicating that the hydrogel is capable of anti-inflammation in the ICH mouse model. In general, proinflammatory cytokines are thought to be produced mainly by activated M1 microglia/macrophages in the brain (Bai et al., 2020), Our results have shown that the hydrogel injection can inhibit the M1 phenotype, which might explain the decreased expression of the IL-1β and TNF-α.

### Gelatin Hydrogel Injection Upregulated the Integrin β1 Expression

**FIGURE 8:**
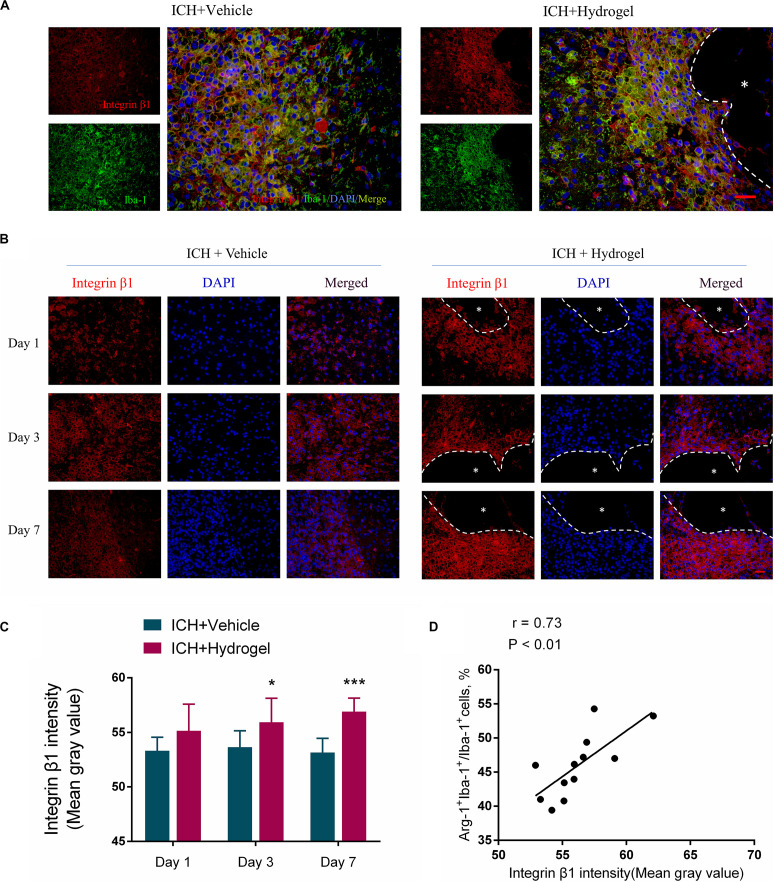
Gelatin hydrogel affects the expression of integrin β1. **(A)** Double immunofluorescence staining was performed with integrin β1 (red) and microglia marker/macrophages Iba-1 (green) in brain sections on day 7, scale bar = 20 μm. **(B)** Immunofluorescence staining of integrin β1 around the lesion, scale bar = 20 μm. **(C)** Fluorescence intensity (FI) analysis of the images. **(D)** The correlation analysis between the Arg^+^IBA^+^ double-positive cells positive percentage and fluorescence intensity of β1, the correlation coefficient is 0.73 (*P* < 0.01). *Represents the hydrogel. Data are mean ± SD, *n* = 4 mice per group, *P < 0.05, ***P < 0.001.

The injectable gelatin hydrogel contains cell adhesive motifs-RGD, which is a ligand of the integrin. Previous studies have found that the integrin recruitment was elevated by RGD adhesive motifs to aid the formation of adhesive structures, and the β1, an integrin subunit, mediated interactions can control macrophage polarization and promote Schwann cells migration *in vitro* (Zhang et al., 2016; Cha et al., 2017; Kang et al., 2019). Therefore, to examine whether the difference of microglial/macrophages phenotype in two groups might result from downstream signaling regulation via integrin, the expression of β1 was detected by fluorescence intensity using immunofluorescence staining. Double staining was performed at first to localize the β1, and the results indicated that the β1 was mainly expressed in the activated Iba-1 positive microglia/macrophages around the lesion (Figure 8A), which consistent previous report that β1 was expressed on the microglia in the CNS (Milner and Campbell, 2002). Next, the expression of β1 in two groups was compared, and it was shown that the expression of β1 in the ICH + Hydrogel group exhibited an increasing tendency, while the one was decreased gradually with time in the ICH + Vehicle group, and showed a statistical difference on day 3 and 7 (Figures 8B,C), indicating that the injectable gelatin hydrogel is capable of upregulating the β1 expression around the lesion in the brain after ICH.

Interestingly, our results also showed that the expression of β1 in the two groups was consistent with the changing trend of the Arg + Iba-1 + double-positive cells. We further analyzed the correlation between the trends of these two parameters. It was found that both parameters correlated well, with a correlation coefficient of 0.73 (*p* < 0.01) in the ICH + Hydrogel group (Figure 8D), which means, the expression of integrin β1 was positively correlated with the percentage of the Arg-1 + Iba-1 + cells, while the expression of integrin β1 in the ICH + Vehicle group did not change significantly over time and the percentage of the Arg-1 + Iba-1 + cells remained stable, so it is tempting to speculate that integrin β1 appears to play an important role in microglia/macrophages polarization.

### Mechanism Discussion

**FIGURE 9:**
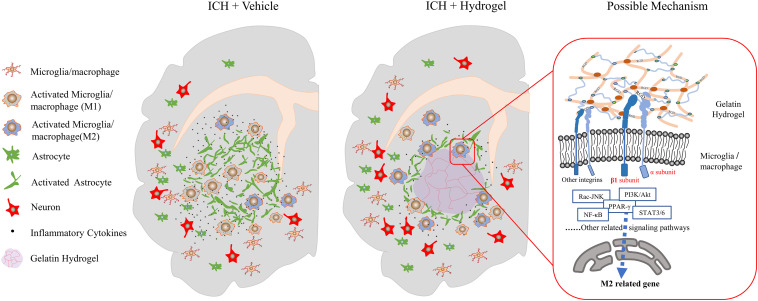
Schematic presentation of the effect of the injectable gelatin hydrogel on the host cells and proposed mechanism.

In this study, injectable gelatin hydrogel displays the interaction with different host cells such as decreasing the astrocytes activation, reducing the neuronal loss, especially affecting the microglia/macrophages response, which makes it possible to suppress inflammation and enhance functional recovery (Figure 9). However, there is no clear idea about the reasons to explain this phenomenon. The polarization of microglia/macrophages is a complex multi-factor interaction process that regulated by a variety of intracellular signaling molecules and pathways, e.g., JNK, PI3K/Akt, Notch, and JAK/STAT signaling pathway (Zhou et al., 2014).

Integrin was found to be involved in the polarization of microglia/macrophages through multiple signaling pathways. For example, in the tumor microenvironment, interstitial flow through the integrin β1 promotes autophosphorylation of FAK, which activates downstream Src/Akt, and transmits the signal to STAT3/6, thus promoting the M2-type polarization of macrophages (Li et al., 2018). In the pulmonary fibrosis model, the extracellular matrix via the α4β1 integrin leads to the activation of Rac2 and potentially regulates macrophage M2 differentiation (Joshi et al., 2017). In the colitis model, the Integrin αVβ3 polarized macrophages toward M1-type by promoting the overactivation of STAT1/3 downstream of the ILK/Akt/mTOR signaling pathway (Piedra-Quintero et al., 2019). *In vitro* environment of hydrogels containing RGD, with the presence of interleukin-4, integrin α2β1 may cause the polarization of macrophages to M2 through STAT6 and to M1 through IRF5 (Cha et al., 2017).

Our study revealed that, after the gelatin hydrogel injection, the pro-inflammatory M1 microglia/macrophages polarization was suppressed while the anti-inflammatory M2 phenotype was promoted, and the secretion of inflammatory cytokines was reduced, which was accompanied by the upregulation of integrin β1. Therefore, integrinβ1 appeared to play an important role in the negative regulation of neuroinflammation in our condition. However, the mechanism in this process still needs further verification. Meanwhile, other integrins’ interaction with different ligands may also have significant effects on the microglia/macrophages (Plow et al., 2000). Here, a mechanism was proposed as follows based on literature and our results.

When the gelatin hydrogel was injected into the lesion site, the RGD sequences along the molecular chains were steadily exposed, which would bind to and promote the expression of the integrins on the surface of the surrounding cells such as microglia/macrophages around the lesion. Due to the acceptor-ligand interactions, the binding integrins activated the FAK-mediated signal transduction through the M2-associated signal pathways, e.g., FAK-PI3K-AKT/Rac-JNK, or FAK-STAT3/6, and then the signals were transmitted to transcription factors(NF-κB, SOCS, or PPAR-γ) and promoted the M2 phenotype polarization of the microglia/macrophages (Figure 9).

Overall, our study suggested that the integrin might play an important role in the polarization of microglia/macrophages after the gelatin hydrogel injection, and therefore systematical analysis of the role of the integrin family on microglia/macrophages could provide the fundamental basis for creating next-generation biomaterials that controllably induce M1 or M2 microglia/macrophages polarization.

## Conclusion

In conclusion, we successfully synthesized the thiolated gelatin and prepared the injectable gelatin hydrogel. The hydrogel was characterized by SEM, porosity, rheology, and cytotoxicity *in vitro*, and then evaluated in a mouse model of ICH. The *in vivo* study indicated that the hydrogel injection could reduce the neuron loss, promote the nerve functional recovery, decrease the activation of the microglia/macrophages and astrocytes, and modulate microglia/macrophages polarization to decrease the release of the inflammatory cytokines such as IL-1β and TNF-α. The related mechanism was proposed and discussed, which would provide the basis of new design concepts for the biomaterials that can directly suppress inflammation.

## Data Availability Statement

The original contributions presented in the study are included in the article/Supplementary Material, further inquiries can be directed to the corresponding author.

## Ethics Statement

The animal study was reviewed and approved by the Ethics Committee of Sichuan University.

## Author Contributions

JX performed the experiments and wrote the manuscript and the discussion of the results. ZD wrote the part of the manuscript and the discussion of the results. XQ, YO, LZ, and YW performed the part of experiments. XG, LM, HLiu, HLi, and CY were involved in the discussion of the results. MT was responsible for conceptualizing, performing the experiments, the discussion of the results, and revising the manuscript. All authors contributed to the article and approved the submitted version.

## Conflict of Interest

The authors declare that the research was conducted in the absence of any commercial or financial relationships that could be construed as a potential conflict of interest.

## Footnotes

1 https://imagej.nih.gov/ij/

## References

[B1] Adams K. L. Gallo V. (2018). The diversity and disparity of the glial scar. *Nat. Neurosci.* 21 9–15. 10.1038/s41593-017-0033-9 29269757 PMC5937232

[B2] Anderson M. A. Burda J. E. Ren Y. Ao Y. O’Shea T. M. Kawaguchi R. (2016). Astrocyte scar formation aids central nervous system axon regeneration. *Nature* 532 195–200. 10.1038/nature17623 27027288 PMC5243141

[B3] Bai Q. Xue M. Yong V. W. (2020). Microglia and macrophage phenotypes in intracerebral haemorrhage injury: therapeutic opportunities. *Brain* 143 1297–1314. 10.1093/brain/awz393 31919518

[B4] Barthes J. Dollinger C. Muller C. B. Liivas U. Dupret-Bories A. Knopf-Marques H. (2018). Immune assisted tissue engineering via incorporation of macrophages in cell-laden hydrogels under cytokine stimulation. *Front. Bioeng. Biotechnol.* 6:108. 10.3389/fbioe.2018.00108 30177966 PMC6110199

[B5] Budday S. Sommer G. Birkl C. Langkammer C. Haybaeck J. Kohnert J. (2017). Mechanical characterization of human brain tissue. *Acta Biomater.* 48 319–340. 10.1016/j.actbio.2016.10.036 27989920

[B6] Butovsky O. Jedrychowski M. P. Moore C. S. Cialic R. Lanser A. J. Gabriely G. (2014). Identification of a unique TGF-β dependent molecular and functional signature in microglia. *Nat. Neurosci.* 17 131–143. 10.1038/nn.3599 24316888 PMC4066672

[B7] Cha B.-H. Shin S. R. Leijten J. Li Y.-C. Singh S. Liu J. C. (2017). Integrin-mediated interactions control macrophage polarization in 3D hydrogels. *Adv. Healthcare Mater.* 6:1700289. 10.1002/adhm.201700289 28782184 PMC5677560

[B8] Chen Z. Xu N. Dai X. Zhao C. Wu X. Shankar S. (2019). Interleukin-33 reduces neuronal damage and white matter injury via selective microglia M2 polarization after intracerebral hemorrhage in rats. *Brain Res. Bull.* 150 127–135. 10.1016/j.brainresbull.2019.05.016 31129170

[B9] Cregg J. M. DePaul M. A. Filous A. R. Lang B. T. Tran A. Silver J. (2014). Functional regeneration beyond the glial scar. *Exp. Neurol.* 253 197–207. 10.1016/j.expneurol.2013.12.024 24424280 PMC3951813

[B10] Dimatteo R. Darling N. J. Segura T. (2018). In situ forming injectable hydrogels for drug delivery and wound repair. *Adv. Drug Deliv. Rev.* 127 167–184. 10.1016/j.addr.2018.03.007 29567395 PMC6003852

[B11] Doyle K. P. Simon R. P. Stenzel-Poore M. P. (2008). Mechanisms of ischemic brain damage. *Neuropharmacology* 55 310–318. 10.1016/j.neuropharm.2008.01.005 18308346 PMC2603601

[B12] Duan Z. Li H. Qi X. Wei Y. Guo X. Li Y. (2019). Crocin attenuation of neurological deficits in a mouse model of intracerebral hemorrhage. *Brain Res. Bull.* 150 186–195. 10.1016/j.brainresbull.2019.05.023 31173858

[B13] Dursun U. T. Yucel D. Hasirci V. (2019). A novel GelMA-pHEMA hydrogel nerve guide for the treatment of peripheral nerve damages. *Int. J. Biol. Macromol.* 121 699–706. 10.1016/j.ijbiomac.2018.10.060 30336245

[B14] Echave M. C. Saenz del Burgo L. Pedraz J. L. Orive G. (2017). Gelatin as biomaterial for tissue engineering. *Curr. Pharm. Des.* 23 3567–3584. 10.2174/0929867324666170511123101 28494717

[B15] Engler A. J. Sen S. Sweeney H. L. Discher D. E. (2006). Matrix elasticity directs stem cell lineage specification. *Cell* 126 677–689. 10.1016/j.cell.2006.06.044 16923388

[B16] Feng X. Xu W. Li Z. Song W. Ding J. Chen X. (2019). Immunomodulatory nanosystems. *Adv. Sci.* 6:1900101. 10.1002/advs.201900101 31508270 PMC6724480

[B17] Gao Z. Min C. Xie H. Qin J. He X. Zhou S. (2020). TNFR2 knockdown triggers apoptosis-induced proliferation in primarily cultured Schwann cells. *Neurosci. Res.* 150 29–36. 10.1016/j.neures.2019.01.010 30731109

[B18] Ge L. You X. Huang K. Kang Y. Chen Y. Zhu Y. (2018). Screening of novel RGD peptides to modify nanoparticles for targeted cancer therapy. *Biomater. Sci.* 6 125–135. 10.1039/c7bm00776k 29142995

[B19] Ghuman H. Massensini A. R. Donnelly J. Kim S.-M. Medberry C. J. Badylak S. F. (2016). ECM hydrogel for the treatment of stroke: characterization of the host cell infiltrate. *Biomaterials* 91 166–181. 10.1016/j.biomaterials.2016.03.014 27031811 PMC4893791

[B20] Ghuman H. Mauney C. Donnelly J. Massensini A. R. Badylak S. F. Modo M. (2018). Biodegradation of ECM hydrogel promotes endogenous brain tissue restoration in a rat model of stroke. *Acta Biomater.* 80 66–84. 10.1016/j.actbio.2018.09.020 30232030 PMC6217851

[B21] Gorenkova N. Osama I. Seib F. P. Carswell H. V. O. (2019). In vivo evaluation of engineered self-assembling silk fibroin hydrogels after intracerebral injection in a rat stroke model. *ACS Biomater. Sci. Eng.* 5 859–869. 10.1021/acsbiomaterials.8b01024 33405845

[B22] Guo X. Qi X. Li H. Duan Z. Wei Y. Zhang F. (2019). Deferoxamine alleviates iron overload and brain injury in a rat model of brainstem hemorrhage. *World Neurosurg.* 128 e895–e904. 10.1016/j.wneu.2019.05.024 31082547

[B23] Hazel K. (1998). Citicoline treatment for experimental intracerebral hemorrhage in mice editorial comment. *Stroke* 29 2136–2140. 10.1161/01.STR.29.10.2136 9756595

[B24] Hu Y. (2016). Preclinical studies of stem cell transplantation in intracerebral hemorrhage: a systemic review and meta-analysis. *Mol. Neurobiol.* 9 5269–5267. 10.1007/s12035-015-9441-6 PMC5012148 26409481

[B25] Huettner N. Dargaville T. R. Forget A. (2018). Discovering cell-adhesion peptides in tissue engineering: beyond RGD. *Trends Biotechnol.* 36 372–383. 10.1016/j.tibtech.2018.01.008 29422411

[B26] Jin Y.-C. Lee H. Kim S.-W. Kim I.-D. Lee H.-K. Lee Y. (2016). Intranasal delivery of RGD motif-containing osteopontin icosamer confers neuroprotection in the postischemic brain via αvβ3 integrin binding. *Mol. Neurobiol.* 53 5652–5663. 10.1007/s12035-015-9480-z 26482372

[B27] Joshi S. Singh A. R. Wong S. S. Zulcic M. Jiang M. Pardo A. (2017). Rac2 is required for alternative macrophage activation and bleomycin induced pulmonary fibrosis; a macrophage autonomous phenotype. *PLoS One* 12:e0182851. 10.1371/journal.pone.0182851 28817691 PMC5560537

[B28] Kang H. Wong S. H. D. Pan Q. Li G. Bian L. (2019). Anisotropic ligand nanogeometry modulates the adhesion and polarization state of macrophages. *Nano Lett.* 19 1963–1975. 10.1021/acs.nanolett.8b05150 30740982

[B29] Krishnamurthi R. V. Feigin V. L. Forouzanfar M. H. Mensah G. A. Connor M. Bennett D. A. (2013). Global and regional burden of first-ever ischaemic and haemorrhagic stroke during 1990–2010: findings from the global burden of disease study 2010. *Lancet Global Health* 1 e259–e281. 10.1016/S2214-109X(13)70089-5 25104492 PMC4181351

[B30] Lan X. Han X. Li Q. Yang Q.-W. Wang J. (2017). Modulators of microglial activation and polarization after intracerebral haemorrhage. *Nat. Rev. Neurol.* 13 420–433. 10.1038/nrneurol.2017.69 28524175 PMC5575938

[B31] Li R. Serrano J. C. Xing H. Lee T. A. Azizgolshani H. Zaman M. (2018). Interstitial flow promotes macrophage polarization toward an M2 phenotype. *Mol. Biol. Cell* 29 1927–1940. 10.1091/mbc.E18-03-0164 29995595 PMC6232969

[B32] Li S. Nih L. R. Bachman H. Fei P. Li Y. Nam E. (2017). Hydrogels with precisely controlled integrin activation dictate vascular patterning and permeability. *Nat. Mater* 16 953–961. 10.1038/nmat4954 28783156 PMC5809173

[B33] Lin J. Ding J. Dai Y. Wang X. Wei J. Chen Y. (2017). Antibacterial zinc oxide hybrid with gelatin coating. *Mater. Sci. Eng. C* 81 321–326. 10.1016/j.msec.2017.08.009 28887979

[B34] Lynn A. D. Kyriakides T. R. Bryant S. J. (2010). Characterization of the in vitro macrophage response and in vivo host response to poly(ethylene glycol)-based hydrogels. *J. Biomed. Mater. Res. A* 93 941–953. 10.1002/jbm.a.32595 19708075

[B35] Miao H. Runming L. Cong H. Xiuzhen L. Hang Z. (2018). Minocycline promotes post-hemorrhagic neurogenesis via M2 microglia polarization via up-regulation of the TrkB/BDNF pathway in rats. *J. Neurophysiol.* 120 1307–1317. 10.1152/jn.00234.2018 29790836

[B36] Michael M. Parsons M. (2020). New perspectives on integrin-dependent adhesions. *Curr. Opin. Cell Biol.* 63 31–37. 10.1016/j.ceb.2019.12.008 31945690 PMC7262580

[B37] Milner R. Campbell I. L. (2002). The integrin family of cell adhesion molecules has multiple functions within the CNS. *J. Neurosci. Res.* 69 286–291. 10.1002/jnr.10321 12125070

[B38] Mobaraki M. Abbasi R. Vandchali S. O. Ghaffari M. Moztarzadeh F. Mozafari M. (2019). Corneal repair and regeneration: current concepts and future directions. *Front. Bioeng. Biotechnol.* 7:135. 10.3389/fbioe.2019.00135 31245365 PMC6579817

[B39] Modo M. (2019). Bioscaffold-induced brain tissue regeneration. *Front. Neurosci.* 13:1156. 10.3389/fnins.2019.01156 31787865 PMC6855095

[B40] Möller B. Villiger P. M. (2006). Inhibition of IL-1, IL-6, and TNF-α in immune-mediated inflammatory diseases. *Springer Semin. Immun.* 27 391–408. 10.1007/s00281-006-0012-9 16738952

[B41] Motamed S. Del Borgo M. P. Zhou K. Kulkarni K. Crack P. J. Merson T. D. (2019). Migration and differentiation of neural stem cells diverted from the subventricular zone by an injectable Self-assembling β-Peptide hydrogel. *Front. Bioeng Biotechnol.* 7:315. 10.3389/fbioe.2019.00315 31788470 PMC6856563

[B42] Nguyen V.-T. Ko S.-C. Oh G.-W. Heo S.-Y. Jeon Y.-J. Park W. S. (2016). Anti-inflammatory effects of sodium alginate/gelatine porous scaffolds merged with fucoidan in murine microglial BV2 cells. *Int. J. Biol. Macromol.* 93 1620–1632. 10.1016/j.ijbiomac.2016.05.078 27234497

[B43] Nih L. R. Carmichael S. T. Segura T. (2016). Hydrogels for brain repair after stroke: an emerging treatment option. *Curr. Opin. Biotechnol.* 40 155–163. 10.1016/j.copbio.2016.04.021 27162093 PMC4975623

[B44] Nih L. R. Gojgini S. Carmichael S. T. Segura T. (2018). Dual-function injectable angiogenic biomaterial for the repair of brain tissue following stroke. *Nat. Mater* 17 642–651. 10.1038/s41563-018-0083-8 29784996 PMC6019573

[B45] Nih L. R. Sideris E. Carmichael S. T. Segura T. (2017). Injection of microporous annealing particle (MAP) hydrogels in the stroke cavity reduces gliosis and inflammation and promotes NPC migration to the lesion. *Adv. Mater.* 29:1606471. 10.1002/adma.201606471 28650574 PMC5595584

[B46] Piedra-Quintero Z. L. Serrano C. Villegas-Sepúlveda N. Maravillas-Montero J. L. Romero-Ramírez S. Shibayama M. (2019). Myosin 1F regulates M1-Polarization by stimulating intercellular adhesion in macrophages. *Front. Immunol.* 9:3118. 10.3389/fimmu.2018.03118 30687322 PMC6335276

[B47] Plow E. F. Haas T. A. Zhang L. Loftus J. Smith J. W. (2000). Ligand binding to integrins. *J. Biol. Chem.* 275 21785–21788. 10.1074/jbc.R000003200 10801897

[B48] Poon M. T. C. Fonville A. F. Al-Shahi Salman R. (2014). Long-term prognosis after intracerebral haemorrhage: systematic review and meta-analysis. *J. Neurol. Neurosurg. Psychiatry* 85 660–667. 10.1136/jnnp-2013-306476 24262916

[B49] Rajkovic O. Potjewyd G. Pinteaux E. (2018). Regenerative medicine therapies for targeting neuroinflammation after stroke. *Front. Neurol.* 9:734. 10.3389/fneur.2018.00734 30233484 PMC6129611

[B50] Rowley A. T. Nagalla R. R. Wang S.-W. Liu W. F. (2019). Extracellular matrix-based strategies for immunomodulatory biomaterials engineering. *Adv. Healthc. Mater.* 8:1801578. 10.1002/adhm.201801578 30714328 PMC7568845

[B51] Samadian H. Maleki H. Fathollahi A. Salehi M. Gholizadeh S. Derakhshankhah H. (2020). Naturally occurring biological macromolecules-based hydrogels: potential biomaterials for peripheral nerve regeneration. *Int. J. Biol. Macromol.* 154 795–817. 10.1016/j.ijbiomac.2020.03.155 32198035

[B52] Schaar K. L. Brenneman M. M. Savitz S. I. (2010). Functional assessments in the rodent stroke model. *Exp. Transl. Stroke Med.* 2:13. 10.1186/2040-7378-2-13 20642841 PMC2915950

[B53] Shi C. Bi C. Ding M. Xie J. Xu C. Qiao R. (2019a). Polymorphism and stability of nanostructures of three types of collagens from bovine flexor tendon, rat tail, and tilapia skin. *Food Hydrocolloids* 93 253–260. 10.1016/j.foodhyd.2019.02.035

[B54] Shi C. He Y. Ding M. Wang Y. Zhong J. (2019b). Nanoimaging of food proteins by atomic force microscopy. Part II: application for food proteins from different sources. *Trends Food Sci. Technol.* 87 14–25. 10.1016/j.tifs.2018.11.027

[B55] Shu X. Z. Liu Y. Palumbo F. Prestwich G. D. (2003). Disulfide-crosslinked hyaluronan-gelatin hydrogel films: a covalent mimic of the extracellular matrix for in vitro cell growth. *Biomaterials* 24 3825–3834. 10.1016/S0142-9612(03)00267-9 12818555

[B56] Taraballi F. Sushnitha M. Tsao C. Bauza G. Liverani C. Shi A. (2018). Biomimetic tissue engineering: tuning the immune and inflammatory response to implantable biomaterials. *Adv. Healthc Mater.* 7:e1800490. 10.1002/adhm.201800490 29995315

[B57] Tian M. Yang Z. Kuwahara K. Nimni M. E. Wan C. Han B. (2012). Delivery of demineralized bone matrix powder using a thermogelling chitosan carrier. *Acta Biomater.* 8 753–762. 10.1016/j.actbio.2011.10.030 22079781

[B58] Tschoe C. Bushnell C. D. Duncan P. W. Alexander-Miller M. A. Wolfe S. Q. (2020). Neuroinflammation after intracerebral hemorrhage and potential therapeutic targets. *J. Stroke* 22 29–46. 10.5853/jos.2019.02236 32027790 PMC7005353

[B59] Tsui C. Koss K. Churchward M. A. Todd K. G. (2019). Biomaterials and glia: progress on designs to modulate neuroinflammation. *Acta Biomater.* 83 13–28. 10.1016/j.actbio.2018.11.008 30414483

[B60] Wang C. Feng N. Chang F. Wang J. Yuan B. Cheng Y. (2019). Injectable cholesterol-enhanced stereocomplex polylactide thermogel loading chondrocytes for optimized cartilage regeneration. *Adv. Healthc. Mater.* 8:1900312. 10.1002/adhm.201900312 31094096

[B61] Wang Y. Jiang Z. Xu W. Yang Y. Zhuang X. Ding J. (2019). Chiral polypeptide thermogels induce controlled inflammatory response as potential immunoadjuvants. *ACS Appl. Mater. Interfaces* 11 8725–8730. 10.1021/acsami.9b01872 30785721

[B62] Wang H. Morales R. T. Cui X. Huang J. Qian W. Tong J. (2018). A photoresponsive hyaluronan hydrogel nanocomposite for dynamic macrophage immunomodulation. *Adv. Healthc. Mater.* 8:e1801234. 10.1002/adhm.201801234 30537061 PMC6392032

[B63] Wang J. Doré S. (2007). Inflammation after intracerebral hemorrhage. *J. Cereb. Blood Flow Metab.* 27 894–908. 10.1038/sj.jcbfm.9600403 17033693

[B64] Wissing T. B. Bonito V. Haaften E. E. van Doeselaar M. van Brugmans M. M. C. P. Janssen H. M. (2019). Macrophage-driven biomaterial degradation depends on scaffold microarchitecture. *Front. Bioeng. Biotechnol.* 7:87. 10.3389/fbioe.2019.00087 31080796 PMC6497794

[B65] Wu T. Ding M. Shi C. Qiao Y. Wang P. Qiao R. (2020). Resorbable polymer electrospun nanofibers: history, shapes and application for tissue engineering. *Chin. Chem. Lett.* 31 617–625. 10.1016/j.cclet.2019.07.033

[B66] Xiong X.-Y. Liu L. Yang Q.-W. (2016). Functions and mechanisms of microglia/macrophages in neuroinflammation and neurogenesis after stroke. *Prog. Neurobiol.* 142 23–44. 10.1016/j.pneurobio.2016.05.001 27166859

[B67] Yang J. Li Q. Wang Z. Qi C. Han X. Lan X. (2017). Multimodality MRI assessment of grey and white matter injury and blood-brain barrier disruption after intracerebral haemorrhage in mice. *Sci. Rep.* 7:40358. 10.1038/srep40358 28084426 PMC5234017

[B68] Zaveri T. D. Lewis J. S. Dolgova N. V. Clare-Salzler M. J. Keselowsky B. G. (2014). Integrin-directed modulation of macrophage responses to biomaterials. *Biomaterials* 35 3504–3515. 10.1016/j.biomaterials.2014.01.007 24462356 PMC3970928

[B69] Zhang L. Schallert T. Zhang Z. G. Jiang Q. Arniego P. Li Q. (2002). A test for detecting long-term sensorimotor dysfunction in the mouse after focal cerebral ischemia. *J. Neurosci. Methods* 117 207–214. 10.1016/s0165-0270(02)00114-0 12100987

[B70] Zhang Y. Yu J. Ren K. Zuo J. Ding J. Chen X. (2019). Thermosensitive hydrogels as scaffolds for cartilage tissue engineering. *Biomacromolecules* 20 1478–1492. 10.1021/acs.biomac.9b00043 30843390

[B71] Zhang Z. Yu B. Gu Y. Zhou S. Qian T. Wang Y. (2016). Fibroblast-derived tenascin-C promotes Schwann cell migration through β1-integrin dependent pathway during peripheral nerve regeneration. *Glia* 64 374–385. 10.1002/glia.22934 26497118

[B72] Zhou D. Huang C. Lin Z. Zhan S. Kong L. Fang C. (2014). Macrophage polarization and function with emphasis on the evolving roles of coordinated regulation of cellular signaling pathways. *Cell. Signal.* 26 192–197. 10.1016/j.cellsig.2013.11.004 24219909

[B73] Zhou W.-C. Tan P.-F. Chen X.-H. Cen Y. You C. Tan L. (2019). Berberine-incorporated shape memory fiber applied as a novel surgical suture. *Front. Pharmacol.* 10:1506. 10.3389/fphar.2019.01506 31998123 PMC6962190

