# Supplementary figures and images for: Injectable Gelatin Hydrogel Suppresses Inflammation and Enhances Functional Recovery in a Mouse Model of Intracerebral Hemorrhage
Source: Front Bioeng Biotechnol. 2020 Jul 14;8:785. doi: 10.3389/fbioe.2020.00785 (PMC7371925; doi:10.3389/fbioe.2020.00785)

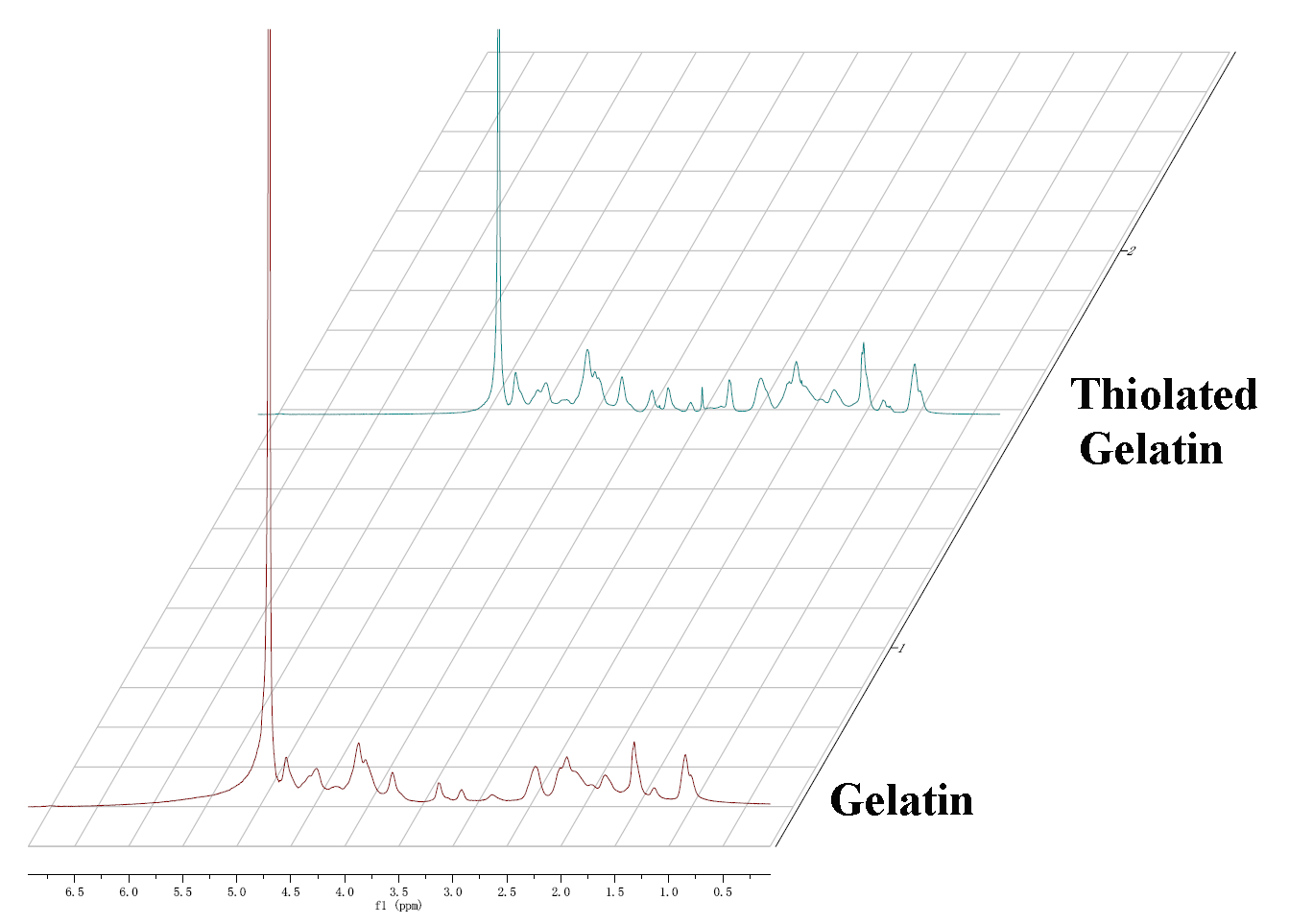


Figure S1. 1HNMR spectra of the gelatin and thiolated gelatin

Supplement: Supplementary file 1 [file Data_Sheet_1.DOCX]
